# External validation of the STUMBL score in a frontline military hospital: predictive performance in conflict-injured patients with thoracic trauma

**DOI:** 10.1186/s12245-025-01072-2

**Published:** 2025-12-29

**Authors:** Ceri Battle, Edward Baker, Jacopo Davide Giamello, Remo Melchio, Dmytro Dmytriiev

**Affiliations:** 1 Physiotherapy Department, Morriston Hospital, Swansea, Wales, UK; 2 Swansea Trials Unit, Swansea University, Swansea, Wales, UK; 3 Emergency Department, Kings College Hospital, London, UK; 4 Department of Emergency Medicine, Santa Croce e Carle Hospital, Cuneo, Italy; 5 School of Emergency Medicine, University of Turin, Cuneo, Italy; 6 Department of Internal Medicine, Santa Croce e Carle Hospital, Cuneo, Italy; 7 Department of Anesthesiology and Intensive Care, Vinnytsia National Medical University, Vinnytsia, Ukraine; 8 Pain Medicine Research Department, Prometei Pain Management and Rehabilitation Centre, Vinnytsia, Ukraine

**Keywords:** Thoracic trauma, Military, STUMBL score, External validation study

## Abstract

**Purpose:**

Thoracic trauma is a frequent cause of emergency department admission in both civilian and military settings however injuries sustained in conflict zones differ in mechanism and severity. The STUMBL Score is a clinical prediction tool originally developed for blunt thoracic trauma in civilian populations. This study aimed to externally validate the STUMBL Score in a wartime context.

**Methods:**

We conducted a retrospective, single-centre validation study of adult patients with thoracic trauma admitted to the ED of a frontline Ukrainian hospital between January 2023 and December 2024. The primary composite outcome was defined as in-hospital mortality, pulmonary complications, or ICU admission. Model performance was assessed by discrimination (area under the receiver operating characteristic curve) and calibration (Hosmer-Lemeshow test and calibration belt).

**Results:**

A total of 374 patients were included (87% male; median age 38 [32–44]). Blast injury was the predominant mechanism. The median STUMBL Score was 30 [24–33], and 92% of patients developed the composite outcome. The area under the receiver operating characteristic curve (AUC) was 0.96 (95% CI 0.94–0.98), and calibration assessed by the Hosmer-Lemeshow test yielded a p-value of 0.812. Using a threshold score of 23, the sensitivity was 0.89 and specificity 0.97.

**Conclusion:**

The STUMBL Score demonstrated excellent predictive performance in a military population with high-acuity, war-related thoracic trauma. These findings support its potential utility in conflict-zone emergency care, although prospective validation in broader military settings is warranted.

**Supplementary Information:**

The online version contains supplementary material available at 10.1186/s12245-025-01072-2.

## Introduction

Thoracic trauma is a frequent cause of emergency department (ED) attendance as a consequence of trauma and ranges from mild injuries that can be managed in an out-of-hospital setting to those patients requiring critical care management [1]. Common mechanisms of injury in civilian populations include falls from standing height, road traffic collisions, falls from above two metres and sporting injuries. It is well-recognised that thoracic trauma may be difficult to manage, due to the delayed onset of complications [2, 3]. The identification of patients at high risk of developing delayed complications is challenging, and a number of clinical prediction models have been developed to assist in clinician decision-making in the ED [4].

One such model is the STUMBL (STUdy of the Management of BLunt chest wall trauma) Score, which was originally derived and validated in the United Kingdom (UK) in 2014 for civilian patients with blunt thoracic trauma and comprises five variables including patient age, number of rib fractures, chronic lung disease, use of pre-injury anticoagulants and oxygen saturation levels [5]. When totalled, these variables have been shown to correlate with risk of thoracic trauma-related complications, from which cut-off values can be used to guide discharge disposition from the ED. Whilst originally developed to guide discharge disposition, more recent uses for the model include referral to the acute pain team or physiotherapy, and need for regional analgesia [4]. The STUMBL score has shown good predictive capabilities for patients based on the risk of developing blunt thoracic trauma-related complications, and has been externally validated in a number of different settings, including New Zealand [6], Italy [7] and Australia [8].

The ongoing military conflict in Ukraine has led to a significant shift in the mechanisms and patterns of trauma presenting to emergency departments. Unlike civilian trauma, which is often caused by low-velocity mechanisms such as falls or motor vehicle collisions, combat-related injuries are frequently the result of high-energy blasts, explosive devices, and penetrating trauma [9]. These mechanisms tend to produce more complex thoracic injuries, often in younger, previously healthy individuals. In this setting, rapid and accurate risk stratification tools are urgently needed to support clinical decision-making under resource-constrained and high-pressure conditions [10]. Despite the introduction of the STUMBL Score into several Ukrainian hospitals to provide clinicians with a risk of morbidity and mortality, no prior study has assessed its performance in this unique, high-acuity environment. This study aimed to externally validate the STUMBL Score in a wartime context.

## Methods

### Study design

This single-centre retrospective validation study was conducted in the ED of Vinnytsia National Medical University in Central Ukraine. All adult patients presenting to the ED with thoracic trauma between January 2023 and December 2024 were included. Exclusion criteria included an age of less than 18 years and the presence of an immediately life-threatening or concurrent injury, identified during the first medical evaluation and deemed likely of having a significant impact on the patient management and outcome (as defined in the original derivation and validation study) [5].

The TRIPOD (Transparent Reporting of a Multivariable Prediction Model for Individual Prognosis or Diagnosis) statement [11] was used for the completion of this study and a checklist is provided (supplementary material S1). Ethical approval was granted by the Vinnitsya National Medical University (reference: 0023.2025.0003) and registered at the Ukrainian Institute of Scientific and Technical Expertise and Information (registration: 0125U000238).

### Data collection and definition of variables

Data collection was completed using electronic medical records. Baseline data was collected in order describe patient and injury characteristics and management variables. These included details regarding injury sustained, mechanism of injury, past medical history, chest drain insertion and antibiotic use. STUMBL Score variables included; patient age, the number of ribs fractured (defined as the number of fractures, rather than the number of ribs fractured), chronic lung disease (defined as any chronic productive lung condition such as COPD, bronchiectasis or cystic fibrosis), oxygen saturation levels (defined as the first available following admission to ED). The method for scoring each variable is highlighted in Table 1.

**Table 1:** The STUMBL score clinical prediction model

Risk factor	Scoring
Age	1 point per each 10 years of age (starting at age 10)
Number of rib fractures	3 point per rib fracture
Chronic lung disease	5 points
Pre-injury anti-coagulant use	4 points
Oxygen saturation levels	2 points for each 5% decrease in O2 saturation
**Final risk score**	**Probability of Developing Complications** **(Mean ± Standard Deviation)**
0–10	13% ±6
11–15	29% ±6
16–20	52% ±8
21–25	70% ±6
26–30	80% ±6
30+	88% ±7
**Threshold values**	**Suggested management**
0–11	Consider discharge home
12–25	Consider admission to ward
≥ 26	Consider admission to critical care

The primary outcome used was the development of complications, defined by the composite outcome, which included at least one of the following: in-hospital mortality, any pulmonary complications (defined as pulmonary infection, pleural effusion, pneumothorax, haemothorax, pleural empyema) and need for intensive care unit admission. Prolonged hospitalisation (defined as lasting greater than or equal to seven days) was not included in the composite outcome in this study as in the original derivation study [5], due to the potential impact of confounding caused by different types of healthcare system in new patient cohort. This method was used in previous external validation studies [6, 7]. As secondary outcomes, the STUMBL Score’s performance was also assessed on the individual outcomes comprising the composite outcome. The follow-up period used in this study corresponds to the length of hospitalisation following discharge from the ED.

### Sample size

In the original derivation study, 161 of 274 (59%) patients had a complication. As the STUMBL Score has five predictive variables, we required 50 events to validate the rule. Using the one in ten rule, we would require 85 participants, however due to the unknown complication rate in this study’s military cohort, we decided to include at least 300 participants, which should be achieved over the two year period.

### Statistical analysis

Categorical variables are expressed as numbers (percentage) and compared with the Pearson X^2^ test. Continuous variables were expressed as median [25th percentile; 75th percentile] and were compared with the Mann-Whitney test. The predictive capabilities of the STUMBL score was evaluated; discrimination using the c-statistic (equivalent to the area under the receiver operator curve) and calibration using the Hosmer-Lemeshow test and graphically with the calibration belt [12, 13]. The Youden Index was used to calculate the optimal cut-off STUMBL Score value, capable of maximising sensitivity, specificity, positive predictive value and negative predictive values. Statistical significance was set at *p* < 0.05. There was less than 1% missing data in the dataset. Statistical analyses were performed with R software (4.0.0 GUI 1.71 Catalina build) and STATA/IC 14.0 software.

## Results

A total of 374 patients were included in the study, of which 324 (87%) were male. Injury mechanism was a blast injury in all but one of the patients. Median age was 38 [25th; 75th percentile: 32;44] years, with a median number of five [4;7] rib fractures. A total of 58 (15%) patients sustained a blunt thoracic injury. Baseline characteristics are shown in Table 2.

**Table 2:** Baseline characteristics of patients

	Total (*n* = 374)	No complications (*n* = 30)	Complications (*n* = 344)	*p* value	Unadjusted OR (95%CI) / z score
Age	38 (32;44)	33 (31;34)	40 (32;45)	< 0.001	Z score: -3.3
Male	324 (87%)	11 (37%)	313 (91%)	< 0.001	17.4 (7.6–39.9)
Blunt injury	58 (15%)	30 (100%)	28 (8%)	< 0.001	2.1 (1.6–2.7)
Chronic lung disease	38 (10%)	0 (0%)	38 (11%)	0.055	0.9 (0.9-1.0)
Chronic heart disease	81 (22%)	0 (0%)	81 (24%)	0.003	0.9 (0.8–0.9)
Pre-injury anticoagulant use	99 (26%)	8 (27%)	91 (26%)	0.980	0.9 (0.9-1.0)
Number of rib fractures	5 (4;7)	4 (3;4)	6 (4;7)	< 0.001	Z score: -5.5
Bilateral rib fractures	289 (77%)	2 (7%)	287 (83%)	< 0.001	0.7 (0.6–0.8)
Sternal fracture	86 (23%)	0 (0%)	86 (25%)	0.002	0.9 (0.8–0.9)
Chest CT in ED	369 (99%)	30 (100%)	339 (99%)	n/a	n/a
Intercostal drain insertion in ED	261 (70%)	0 (0%)	261 (76%)	< 0.001	0.7 (0.7–0.8)
Antibiotics prescribed in ED	374 (100%)	30 (100%)	344 (100%)	n/a	n/a
STUMBL Score	30 (24;33)	17 (14;18)	30 (25;33)	< 0.001	Z score: -8.4

n (%); median (25^th^; 75^th^ percentile)

The median STUMBL Score was 30 [24;33]. The median hospital length of stay was 28 [22;33] days. 345 (92%) patients developed the composite primary outcome. Table 1 compares patients who developed the primary composite outcome with those who did not. The STUMBL score was significantly different in the two cohorts (17 [14;18] vs. 30 [25;33], *p* < 0.001).

**Fig. 1:**
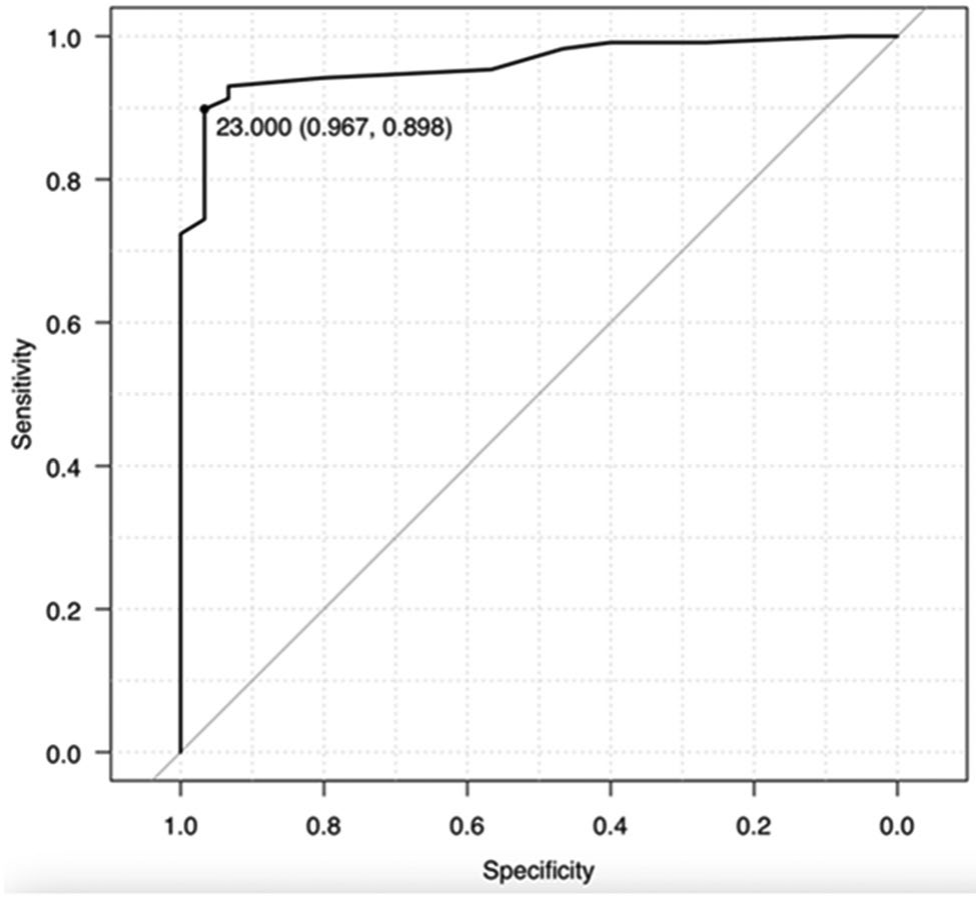
The area under the ROC curve of the STUMBL score

In-hospital mortality was 3%; with 52% of patients requiring intensive care unit admission. The most frequently developed pulmonary complication was pneumonia (85%), followed by haemothorax (82%), pneumothorax (81%), pleural effusion (68%) and empyema (30%). Other complications included wound infections in 14% of patients, and 25% of patients were reported to have hemodynamic instability requiring vasoactive support. Patients admitted to ICU has a median STUMBL score of 31 [28;33]. The area under the ROC curve for the composite outcome was 0.96 (95% CI 0.94–0.98) and is shown in Fig. 1.

**Fig. 2:**
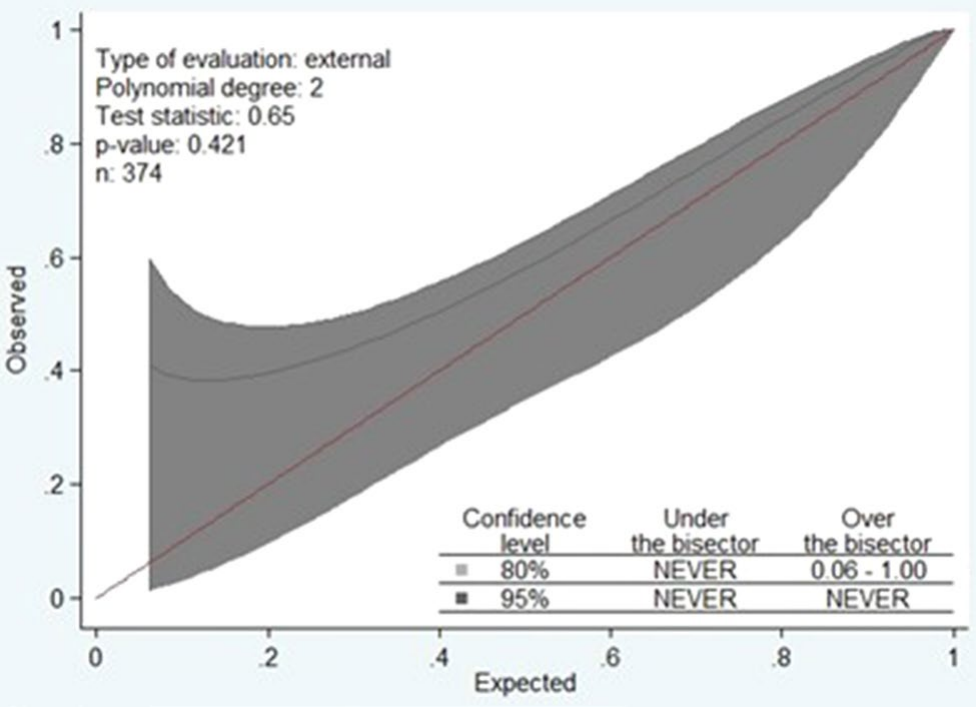
The calibration belt for the STUMBL score

The calibration of the score regarding the composite outcome was evaluated by the Lemeshow test (X-squared = 24.86, *p* = 0.812); in addition, the calibration belt is shown in Fig. 2.

The Youden index has identified the value STUMBL Score of 23 as the cut-off that maximises sensitivity and specificity. This cut-off has a sensitivity of 0.89 (95% CI 0.85,0.92), specificity of 0.97 (95% CI 0.90,1.00), a positive predictive value of 0.99 (95% CI 0.99,1.00), and a negative predictive value of 0.42 (95% CI 0.30,0.54).

## Discussion

This is the first time the STUMBL Score Clinical Prediction Model has been externally validated for a cohort of military patients with thoracic trauma. Penetrating injuries were also included in this study cohort, rather than simply blunt thoracic trauma, on which the STUMBL Score was originally derived. Despite its use in Ukrainian hospitals, the STUMBL Score has not been validated for this cohort, as the original predictor variable number of rib fractures may have less relevance in penetrating trauma. However, the median number of rib fractures sustained by the patients in this study was higher than other external validation studies [6, 7], due to the high number of blast trauma injuries, in which rib fractures are common.

Although the STUMBL Score was originally developed and validated to predict risk of complications and then to guide discharge disposition, the use in this study was to predict whether a patient needed admission to a ward or critical care (without the possibility of home discharge). This is due to the patients being more severely injured, including a penetrating injury, all of whom would be admitted to hospital, regardless of the STUMBL Score. It is worth noting that the complication rate in this study was almost double that reported in the original development study. As a result, the likelihood of complications in this study was therefore higher even at lower STUMBL Score values compared to previous external validation work.

The STUMBL score has been evaluated in a number of previous studies; the original development and validation work first completed in the UK demonstrating a c-statistic of 0.96 (95%CI 0.93–0.98) [5], followed by external validations studies in New Zealand (c-index of 0.73 (95%CI 0.68–0.77)) [6] and most recently, in Italy (c-index of 0.90 (95% CI 0.88–0.93)) [7]. This study demonstrated an c-index of 0.96 (95% CI 0.94–0.98) for the STUMBL Score, based on the optimal cut-off value of 23 derived in our dataset. This demonstrated excellent predictive capabilities in our military cohort of patients with thoracic injuries. The cohort of patients in this study was made up of predominantly younger males, in which a combination of blunt and penetrating thoracic injuries were sustained due to a blast mechanism of injury, leading to a high number of rib fractures and corresponding median STUMBL Score. This is very different to the cohort in the original derivation study, which was older, with a lower median number of rib fractures and corresponding median STUMBL Score due to predominantly falls from a standing height. This is reflected by the primary composite outcome of 92% in this study, compared to 59% in the derivation study [5].

Interestingly, despite the fact that several of the STUMBL Score variables, such as increasing age, chronic respiratory disease and use of pre-injury anticoagulants are less commonly observed in our predominantly young, healthy military population, the model still demonstrated excellent predictive performance. This finding suggests that other components of the score, particularly the number of rib fractures and oxygen saturation levels, may carry greater prognostic weight in high-energy trauma scenarios typical of conflict injuries. It also raises the possibility that the STUMBL Score may be more broadly applicable than previously thought, even in populations with minimal baseline comorbidity. This robustness potentially reinforces its utility in austere environments where rapid, objective clinical decision-making is critical.

The authors of the Italian study suggested that the predictive capabilities of the STUMBL Score in their cohort was higher than that reported in the New Zealand study, due to less heterogeneity in patients’ ethnic groups. The New Zealand authors had proposed this heterogeneity as one of the main differences between their cohort and the original derivation study completed in the UK, leading to lower predictive capabilities in the STUMBL Score in their external validation study. Although not reported in the dataset, the predominant ethnic group of this Ukrainian cohort was ‘White European’, which may have contributed to the improved performance of the STUMBL Score.

In our cohort, the cut-off threshold showing the best sensitivity and specificity was a STUMBL Score of 23, which according to the original derivation study corresponds to a 70% (± 6) risk of experiencing complications. ICU admission is recommended by the STUMBL Score for patients scoring 27 or more. A total of 52% of this cohort were admitted to ICU, which reflects the cohort’s high median STUMBL Score.

Despite the availability of other chest trauma clinical prediction models, the clinical usefulness of the STUMBL score is suggested to be due to its routinely collected variables in the ED setting [14], which may be more apparent in a frontline hospital of a conflict zone. These variables are easily converted into prognostic information regarding the probability of development of thoracic trauma-related complications. This facilitates stratification of patients according to degree of risk, which in turn can potentially lead to a greater accuracy in clinical decision-making. Qualitative research highlights that this is one of the benefits of the STUMBL Score, as reported by Emergency Physicians [14]. 

This study has several limitations. Most importantly, the STUMBL Score was originally developed and validated to guide discharge disposition for patients with blunt chest trauma, whereas this study primarily includes penetrating injuries, most of whom will be admitted to hospital. Until now, the STUMBL Score has not yet been validated for this cohort. The relevance of the original predictor variables included in the STUMBL Score should therefore be considered.

This was a single-centre, retrospective study which may have resulted in a risk of bias, loss of reliability through data input errors and lack of overall generalisability. Other limitations include a lack of detailed data regarding all management interventions, such as analgesia, need for mechanical ventilation or surgical stabilisation of rib fractures. We also lack pre-hospital data and ICU follow-up data. A notable limitation of this study is the extremely high frequency of the composite primary outcome, observed in 92% of the patient cohort. This high event rate may inflate the model’s apparent discriminative performance, particularly the positive predictive value, while limiting the clinical usefulness of the negative predictive value. It also reduces the model’s ability to effectively distinguish between high- and low-risk patients. In imbalanced datasets such as this one, the area under the ROC curve can remain deceptively high even when the model offers limited incremental prognostic information. It should also be noted that sensitivity, specificity and predictive values are all potentially inflated, as they are based on the cut-off derived in this validation study, rather than the original derivation study. Therefore, while the STUMBL Score demonstrated excellent discrimination in our cohort, these findings should be interpreted with caution.

This external validation study has demonstrated that the STUMBL Score clinical prediction model for managing patient with blunt thoracic trauma has excellent predictive capabilities for conflict-injured military patients with thoracic trauma. Further multi-centre, prospective research is required to determine whether the STUMBL Score has both clinical and cost-effectiveness in the management of patients with thoracic trauma. Future studies with more heterogeneous outcome distributions are also needed to further validate its generalisability.

## Supplementary Information

Below is the link to the electronic supplementary material.

Supplementary Material 1

## Data Availability

The data that support the findings of this study are not openly available due to reasons of sensitivity and are available from the corresponding author upon reasonable request.
